# The association between food insecurity and incident type 2 diabetes in Canada: A population-based cohort study

**DOI:** 10.1371/journal.pone.0195962

**Published:** 2018-05-23

**Authors:** Christopher A. Tait, Mary R. L’Abbé, Peter M. Smith, Laura C. Rosella

**Affiliations:** 1 Division of Epidemiology, Dalla Lana School of Public Health, University of Toronto, Toronto, Ontario, Canada; 2 Institute for Clinical Evaluative Sciences, Toronto, Ontario, Canada; 3 Department of Nutritional Sciences, Faculty of Medicine, University of Toronto, Toronto, Ontario, Canada; 4 Institute for Work & Health, Toronto, Ontario, Canada; 5 Department of Epidemiology and Preventive Medicine, Monash University, Melbourne, Australia; 6 Public Health Ontario, Toronto, Ontario, Canada; McMaster University, CANADA

## Abstract

**Background:**

A pervasive and persistent finding is the health disadvantage experienced by those in food insecure households. While clear associations have been identified between food insecurity and diabetes risk factors, less is known about the relationship between food insecurity and incident type 2 diabetes. The objective of this study is to investigate the association between household food insecurity and the future development of type 2 diabetes.

**Methods:**

We used data from Ontario adult respondents to the 2004 Canadian Community Health Survey, linked to health administrative data (n = 4,739). Food insecurity was assessed with the Household Food Security Survey Module and incident type 2 diabetes cases were identified by the Ontario Diabetes Database. Multivariable adjusted Cox proportional hazards models were used to estimate hazard ratios (HRs) and 95% confidence intervals (CIs) for type 2 diabetes as a function of food insecurity.

**Results:**

Canadians in food insecure households had more than 2 times the risk of developing type 2 diabetes compared to those in food secure households [HR = 2.40, 95% CI = 1.17–4.94]. Additional adjustment for BMI attenuated the association between food insecurity and type 2 diabetes [HR = 2.08, 95% CI = 0.99, 4.36].

**Conclusions:**

Our findings indicate that food insecurity is independently associated with increased diabetes risk, even after adjustment for a broad set of measured confounders. Examining diabetes risk from a broader perspective, including a comprehensive understanding of socioeconomic and biological pathways is paramount for informing policies and interventions aimed at mitigating the future burden of type 2 diabetes.

## Introduction

Globally, there are over 200 million people living with type 2 diabetes [1]. Aging populations, steadily increasing obesity rates, increases in sedentary behaviours, and decreases in diabetes-related mortality signal that the global prevalence of type 2 diabetes will continue to grow [2, 3].

In Canada, type 2 diabetes is one of the most prevalent chronic conditions [4] and is the 7^th^ leading cause of mortality [5]. Over the last decade, the prevalence of type 2 diabetes in Canada has increased by 72%, with 11 million Canadians currently living with diabetes or pre-diabetes. This number is expected to rise to 13.9 million (33% of Canadians) by 2026 [6].

Much of the body of literature concerning type 2 diabetes focuses on management and control [7]. Research that is geared towards prevention focuses heavily on the modification of individual risk behaviours, while less attention is given to the broader social determinants of increased type 2 diabetes risk [8, 9].

Household food security is a measure of deprivation us that is not traditionally included in health research. Household food *insecurity* is described as uncertain, insufficient, or inadequate food access, availability, and utilization due to limited financial resources, and the compromised eating patterns and food consumption that may result [10].

Food insecurity has been identified as a significant social and health problem in Canada. In 2004, it was estimated that 9.2% of Canadian households were food insecure [11]. The most recent estimate from 2014 (excluding 2 provinces and 1 territory that opted not to measure food insecurity in 2014) indicates that this number has risen to 12%, representing 3.2 million Canadians [12].

Evidence from cross-sectional studies has shown that those living with type 2 diabetes have a markedly higher prevalence of food insecurity than those without diabetes [13]. Similar studies have also demonstrated the those living in food insecure households are 2–3 times more likely to have diabetes than adults who are food secure, even after controlling for many important risk factors such as income, employment status, anthropometric measures, and lifestyle factors [14, 15].

Limited budgets for those in food insecure households result in purchasing cheaper, high calorie foods, which can contribute to weight gain and an increased risk of many chronic diseases including type 2 diabetes [16, 17]. The lack of access to nutritious foods is not only a risk factor for developing chronic illness, but also compromises the ability to manage health conditions that involve specific dietary regimens [15, 16].

Though literature has supported evidence for an association between food insecurity and type 2 diabetes risk, studies to date have been limited by their cross-sectional design thus lacking the ability to determine whether food insecurity is a risk factor for diabetes, or whether diabetes places individuals at a higher risk for food insecurity. Prospective longitudinal assessment is critical as cross-sectional studies lack the ability to infer the direction of the relationship between food insecurity and type 2 diabetes [14]. For example, the ‘health selection’ hypothesis has been studied, positing that a decline in health status may precede and ultimately cause downward social mobility and a decrease in income [18]. Consequently, this theory presents evidence for reverse causation, by which poor health may precede financial difficulties [19], especially in cases where early age of diagnosis, and thus longer duration of disease, might predispose individuals to being in a food insecure household [20].

Notably, studies in the Canadian population have suggested that food insecurity is a potent marker of nutrition inequity [21], and that food insecurity is a stronger marker of nutritional vulnerability in Canada than it is in the United States [22].

Indications that the nutritional manifestations of food insecurity differ between Canada and the US highlight the need for more thorough investigations into how experiences of food insecurity contribute to future chronic disease risk in each setting. Further, no longitudinal studies investigating this relationship have been carried out in either context representing a significant gap in the literature.

Current estimates of the future health consequences associated with food insecurity are needed to inform health decision-makers of potential areas for upstream intervention to alleviate the burden type 2 diabetes places on the Canadian healthcare system. Data linkages provide a novel opportunity to study this relationship in a prospective, longitudinal, population-based sample. Accordingly, the objectives of this study are to estimate the risk of type 2 diabetes as a function of food insecurity in the Canadian population, and to investigate the extent to which this association may be mediated by obesity.

## Methods

### Data sources and study population

We used data from Ontario adult respondents to Cycle 2.2 of the Canadian Community Health Survey conducted in 2004, deterministically linked to the Ontario Diabetes Database. The Canadian Community Health Survey (CCHS) is a cross-sectional survey administered by Statistics Canada that uses a multi-stage, stratified, clustered probability sample that is representative of 98% of the Canadian population [23]. Those who are full-time members of the Canadian Forces, reside on First Nations Reserves or Crown Lands, are institutionalized, and who reside in certain remote areas reflect the 2% of the population not captured by the survey. Detailed descriptions of the CCHS survey methodology have been published elsewhere [24].

Importantly, CCHS 2.2 was the first national nutrition survey conducted in Canada since the 1970–1972 Nutrition Canada Survey. Accordingly, this study allowed for a detailed consideration and adjustment for dietary patterns that may influence the association under investigation.

All permanent residents of Ontario are covered by a single-payer health insurance system referred to as the Ontario Health Insurance Plan (OHIP). Using OHIP card numbers, every healthcare related encounter is recorded in administrative health databases held by the Institute for Clinical Evaluative Sciences (ICES).

The Ontario Diabetes Database (ODD) is a comprehensive disease registry that contains all physician-diagnosed diabetes cases identified in Ontario [25]. Briefly, the ODD uses the diagnostic criteria of either having two physician service claims recorded in OHIP or one hospital discharge related to diabetes within a 2-year period to identify incident diabetes cases. The ODD has a sensitivity of 86% and a specificity of 97% for the correct classification of individuals with and without type 2 diabetes [26].

We restricted our analyses to individuals ≥ 18 years of age at baseline who were successfully linked to the ODD (n = 5,539). We further excluded pregnant women from the sample to increase the accuracy of body weight measurements (n = 36), prevalent cases of diabetes before respondents’ 2004 CCHS interview date (n = 636), underweight individuals (n = 112), and those who had missing information on food security status (n = 16). After these exclusions, our analytic cohort consists of 4,739 individuals, 2,050 men and 2,689 women.

### Food insecurity

The Household Food Security Survey Module (HFSSM) was introduced in Canada for the first time in CCHS 2.2, using the same questions as those used in the United States [27]. Household food security status was assessed through an 18-item questionnaire in which 10 items were specific to the experiences of adults in the household and 8 items related to the experiences of children under 18.

The experiences of food insecurity captured on this module range from worrying about running out of food before there is more money to buy more, to the inability to afford a balanced diet, to going hungry, missing meals, and in extreme circumstances, not eating for a whole day because of a lack of food or money for food [28].

In coding responses to the HFSSM, any affirmative response was recoded as 1 point [27]. The final score ranged from 0 to 10 for adults and 0 to 8 for children. Total scores of ≤ 1 point were used to classify individuals as food secure whereas total scores of 2 and greater were used to classify individuals as food insecure.

Households were considered food insecure if either adult or child status was food insecure, and only classified as food secure if both adult and child status were food secure, according to the classification framework developed by Health Canada [10]. Further description of food security status classification can be seen in S1 Table.

### Type 2 diabetes

Incident type 2 diabetes cases were identified by the Ontario Diabetes Database which includes information on who was diagnosed in addition to the date of diagnosis. The follow-up period for diabetes cases was calculated as the time from their 2004 CCHS interview to their diabetes diagnosis event. Those who did not develop diabetes over the study period were censored at the end of the observation period (March 31, 2016), or at the time of death if it occurred prior to the end of the observation period. The median follow-up time for our cohort was 11.6 years (range 0.0–12.1 years).

### Covariates

At baseline, participants reported demographic characteristics, health behaviours, and medical histories using self-reported questionnaires and a detailed assessment of food intake through a 24-hour dietary recall. Informed by previous studies investigating food insecurity and type 2 diabetes [13, 14], the following covariates were included for adjustment in our analyses: continuous age in years, gender, income quintiles, race (white/non-white), physical activity, smoking status (current, former, never), alcohol consumption, diet quality, and body mass index (BMI) categories. Missing values were included in the models as dummy variables.

Physical activity was measured by the daily energy expended for leisure time activities, calculated by multiplying the number of times engaged in each type of activity in the past year, average duration of participation in hours, and metabolic equivalent of task (MET) value assigned to each activity [29]. Respondents were categorized as being inactive (0.1–1.4kcal/kg/day), moderately active (1.5–3.0 kcal/kg/day), and active (≥3.0 kcal/kg/day).

Alcohol consumption was categorized according to gender specific cut-offs for the number of alcoholic drinks consumed in the previous week: non-drinker (did not consume alcohol in the last 12 months or drinks less than weekly); occasional drinker (1–3 (men) or 1–2 (women) drinks; regular drinker (4–21 (men) or 3–14 (women) drinks); or heavy drinker (≥21 (men) or ≥14 (women) drinks, or binging behaviour on a weekly basis (≥5 drinks on any occasion).

Diet quality was measured using the Canadian adaptation of the *Healthy Eating Index* (HEI), an *a priori* diet quality index that assesses how congruent respondents’ diets are with Canadian dietary recommendations [30]. Briefly, face-to-face 24-hour dietary recall interviews were conducted on all respondents. A second recall was collected 3–10 days later on a 30% subset of this sample for reliability of the dietary recall. Responses from the dietary recall were scored according to the components of the HEI, which was summed up to an overall diet quality score ranging from 0–100.

BMI was calculated by dividing body weight by the square of body height (kg/m^2^) and classified according to the international standard [31]: underweight (<18.5 kg/m^2^), normal weight (18.5–24.9 kg/m^2^), overweight (25.0–29.9 kg/m^2^), and obese (≥30 kg/m^2^). In CCHS 2.2, BMI measurements were self-reported and/or objectively measured. Where available, objective height and weight measurements were used to calculate BMI (65% of respondents). For participants who only had self-reported measurements, correction equations provided by Statistics Canada were applied to adjust for discrepancies between self-reported and objective measures of BMI. These correction equations adjusting for self reported BMI estimates have been shown to provide more accurate estimates of an individual’s true weight status [32].

### Statistical analysis

Descriptive statistics including means, standard deviations, and the distributions of demographic and lifestyle variables were calculated by food security status. Baseline characteristics were compared between food secure and food insecure participants using *T*-tests for continuous variables and *Chi*-square tests for binary and categorical variables.

Cox proportional hazards models were fit to our data using person-days as the underlying time metric. We estimated multivariable hazard ratios (HRs) and 95% confidence intervals (CIs) for type 2 diabetes associated with food insecurity. Multivariable-adjusted HRs are reported using food secure individuals as the reference category. The proportional hazards assumption was met by modeling the interaction term of food security status and person-time, and no statistically significant interactions were found.

We first fit Model 1 adjusting for age and gender, then Model 2 additionally adjusted for income and race. Model 3 further adjusted for health behaviours and diet quality, and given obesity’s potential role as a mediator of this relationship, we explored the effects of adding BMI to the final model.

Sensitivity analyses were conducted by implementing a 1-year and 2-year wash-out period, in which any diabetes cases diagnosed within the first 1 or 2 years of follow-up were excluded. This approach was taken to assess if the original results may have been influenced by reverse causality in which diabetes was present, but potentially undiagnosed, prior to the assessment of food insecurity status.

Bootstrapped sampling weights provided by Statistics Canada were applied to all analyses to adjust for the complex survey design of CCHS and to produce population-based estimates [23]. All statistical analyses were performed using SAS, version 9.4 (SAS Institute, Cary, NC). Informed written consent was provided by all study participants with regards to sharing their information for linkage with the administrative data. The study received ethical approval through the University of Toronto Health Sciences Research Ethics Board as well as the Research Ethics Board of the Sunnybrook Health Sciences Centre through which ICES is affiliated.

## Results

During 12.1 years of follow-up, there were 577 incident type 2 diabetes cases. Table 1 shows the baseline characteristics of the study participants based on food security status. In general, those who were food insecure tended to be significantly younger, female, non-white, lower in income, and had lower quality diets compared to food secure individuals. Additionally, food insecure participants were more likely to be smokers, less physically active, and obese.

**Table 1:** Baseline characteristics of participants according to food security status, household population aged 18 or older, Ontario (n = 4,739), CCHS 2004 (Cycle 2.2).

	Food Secure (n = 4,462)	Food Insecure (n = 277)	p-value*
Age (Mean, SD)	53.7 (21.6)	41.4 (16.2)	< 0.0001
Gender (% Men)	43.8	34.3	0.0019
Race (% White)	89.9	75.1	< 0.0001
Income			< 0.0001
Quintile 1 (%)	16.6	62.8	
Quintile 2 (%)	18.7	15.5	
Quintile 3 (%)	18.2	10.1	
Quintile 4 (%)	16.6	5.4	
Quintile 5 (%)	20.5	1.4	
Smoking			< 0.0001
Current (%)	21.4	50.2	
Former (%)	29.6	19.1	
Never (%)	49.0	30.7	
Alcohol Consumption			< 0.0001
Non-Drinker (%)	20.4	23.8	
Occasional Drinker (%)	36.5	49.1	
Regular Drinker (%)	27.8	22.8	
Heavy Drinker (%)	15.3	4.7	
Physical Activity			0.4774
Inactive (%)	54.8	58.5	
Moderately Active (%)	25.4	23.1	
Active (%)	19.8	18.4	
Body Mass Index (kg/m^2^)			0.0047
Normal Weight (%)	39.0	34.3	
Overweight (%)	33.1	28.9	
Obese (%)	18.8	27.4	
Diet Quality Score (Mean, SD)	61.4 (13.4)	54.8 (13.5)	< 0.0001

* Chi-square tests were applied to compare characteristics of participants as a function of food security status

Note: Percentages may not add up to 100% because of missing observations and rounding

**Fig 1:**
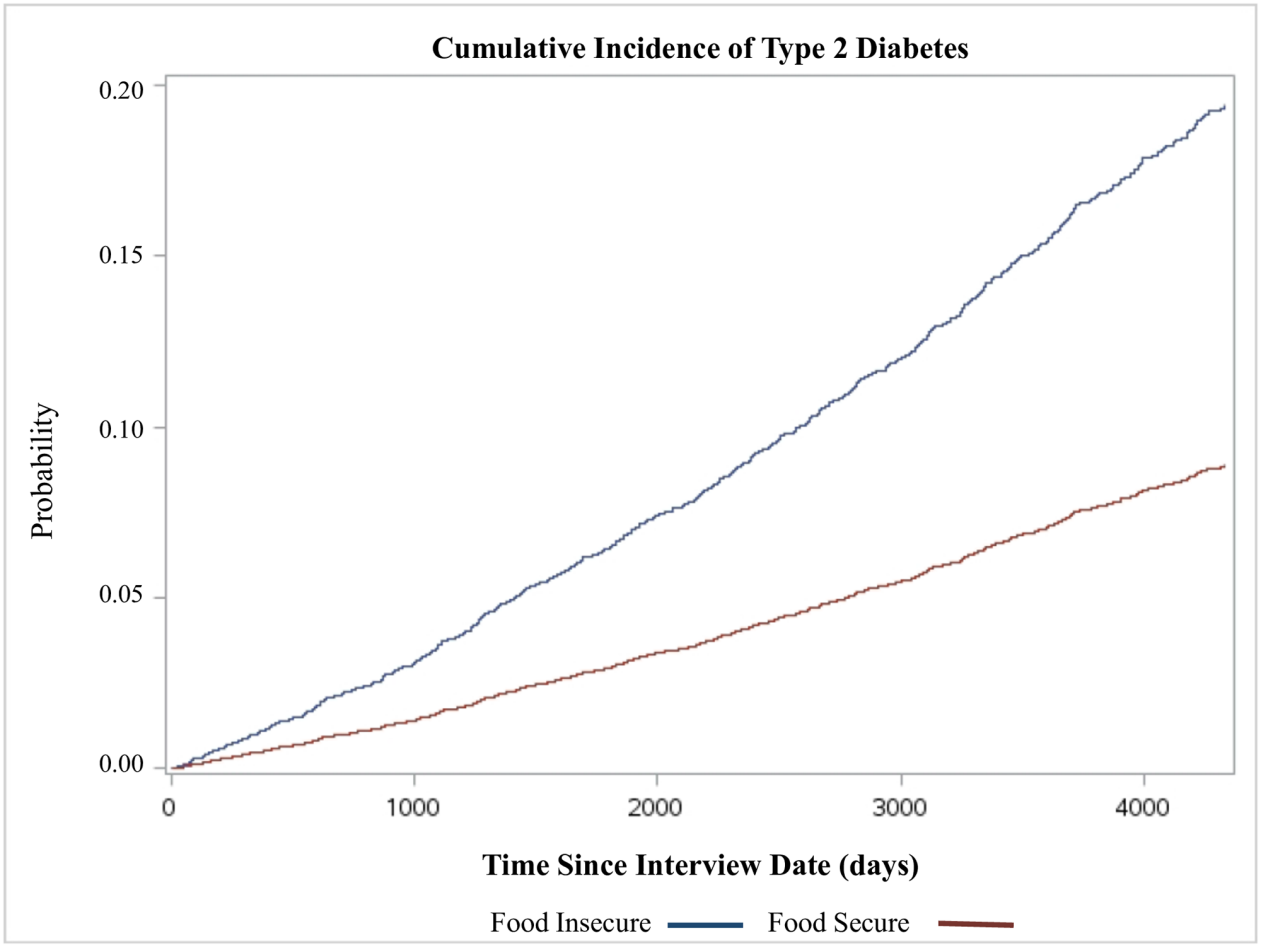
Cumulative incidence of type 2 diabetes by food security status, household population aged 18 or older, Ontario (n = 4,739), CCHS 2004 (Cycle 2.2).

The cumulative incidence of type 2 diabetes for those in food insecure compared to food secure households is shown in Fig 1. In the fully adjusted model (Table 2), Canadians in food insecure households had more than 2 times the risk of type 2 diabetes compared to those in food secure households [HR = 2.40, 95% CI = 1.17–4.94]. Additional adjustment for BMI attenuated the association between food insecurity and type 2 diabetes [HR = 2.08, 95% CI = 0.99, 4.36].

**Table 2:** Multivariable hazard ratios for type 2 diabetes risk by food security status (2004–2016).

Food Security Status	Model 1*		*p-value*	Model 2^†^		*p-value*	Model 3^‡^		*p-value*	Model 4^§^		*p-value*
	Hazard Ratio	95% CI		Hazard Ratio	95% CI		Hazard Ratio	95% CI		Hazard Ratio	95% CI	
Secure	1.00	(referent)		1.00	(referent)		1.00	(referent)		1.00	(referent)	
Insecure	2.31	1.13, 4.70	0.0216	2.47	1.21, 5.06	0.0133	2.40	1.17, 4.94	0.0173	2.08	0.99, 4.36	0.0535

* Model 1 adjusted for age, gender

^†^ Model 2 adjusted for age, gender, income, race

^‡^ Model 3 adjusted for age, gender, income, race, physical activity, smoking, alcohol, diet quality

^§^ Model 4 adjusted for age, gender, income, race, physical activity, smoking, alcohol, diet quality, BMI

As a sensitivity analysis, we excluded individuals who were diagnosed with type 2 diabetes within 1 year and within 2 years of their CCHS interview date respectively (Table 3). These wash out periods allowed us to investigate if reverse causation was influencing our results, given that a diabetes diagnosis date in close proximity to when the exposure was assessed may not accurately reflect the true onset of type 2 diabetes as an event that was preceded by participants’ food security status.

**Table 3:** Sensitivity analyses—Multivariable hazard ratios for type 2 diabetes risk by food security status (2004–2016).

Food Security Status	Model 1*		*p-value*	Model 2^†^		*p-value*	Model 3^‡^		*p-value*	Model 4^§^		*p-value*
	Hazard Ratio	95% CI		Hazard Ratio	95% CI		Hazard Ratio	95% CI		Hazard Ratio	95% CI	
1 Year Wash Out Period (n = 4,682)												
Secure	1.00	(referent)		1.00	(referent)		1.00	(referent)		1.00	(referent)	
Insecure	2.32	1.09, 4.95	0.0296	2.40	1.13, 5.09	0.0231	2.38	1.12, 5.05	0.0238	2.06	0.94, 4.46	0.0694
2 Year Wash Out Period (n = 4,617)												
Secure	1.00	(referent)		1.00	(referent)		1.00	(referent)		1.00	(referent)	
Insecure	2.43	1.06, 5.57	0.0360	2.42	1.06, 5.53	0.0358	2.36	1.03, 5.41	0.0437	2.03	0.86, 4.78	0.1075
Including underweight individuals (n = 4,851)												
Secure	1.00	(referent)		1.00	(referent)		1.00	(referent)		1.00	(referent)	
Insecure	2.30	1.13, 4.70	0.0222	2.47	1.21, 5.06	0.0132	2.40	1.16, 4.94	0.0179	2.07	0.98, 4.34	0.0551

* Model 1 adjusted for age, gender

^†^ Model 2 adjusted for age, gender, income, race

^‡^ Model 3 adjusted for age, gender, income, race, physical activity, smoking, alcohol, diet quality

^§^ Model 4 adjusted for age, gender, income, race, physical activity, smoking, alcohol, diet quality, BMI

After excluding individuals diagnosed within the first year of follow up, the HR and 95% CI did not meaningfully change [HR = 2.38, 95% CI = 1.12, 5.05]. Similar results were seen after excluding individuals diagnosed within the first two years of follow up [HR = 2.36, 95% CI = 1.03, 5.41], suggesting that the results are robust in limiting the potential for reverse causation, although the precision around each point estimate was reduced due to the smaller sample size and number of events.

In re-estimating hazards ratios after re-including underweight individuals in the sample, point estimates were nearly identical to those of the original analysis, indicating that the exclusion of underweight individuals did not influence our results (Table 3).

## Discussion

To our knowledge, this is the first study to investigate the relationship between food insecurity and type 2 diabetes risk in a longitudinal population-based cohort using a validated diabetes registry. Overall, our findings indicate that food insecurity is independently associated with significantly increased diabetes risk, even after adjustment for a comprehensive set of potential confounders. Our findings build upon previous cross-sectional studies, demonstrating the robustness of food insecurity as an independent risk factor for developing type 2 diabetes.

Despite increasing literature on food insecurity and adverse health outcomes, food insecurity has continued to grow in developed countries, suggesting that efforts to stop its growth thus far have been unsuccessful [33, 34]. Food insecurity is often cyclically manifested at the household level, due the nature of monthly pay checks, social assistance, and periodic unforeseen competing financial needs [35]. This cycle of financial instability often contributes to episodic underconsumption, followed by occurrences of overconsumption during times of adequacy, resulting in binge-fast cycles that are associated with insulin resistance and progression to type 2 diabetes [36]. Additionally, the incidence of type 2 diabetes has been directly linked to material deprivation early in life [37], material deprivation later in life [38], as well as more broadly with the stress associated with income, housing, and food insecurity [39].

Notably, evidence has suggested that the mechanism underlying the relationship between food insecurity and diabetes is distinctly different than that between food insecurity and obesity. Repeated episodes of food inadequacy may exacerbate insulin resistance, independently of the pathway through weight gain [14]. Our results also support this finding, given that there is still a large effect of food insecurity on diabetes risk even after adjustment for BMI. This suggests that obesity does not fully explain the pathway through which food insecurity impacts type 2 diabetes risk; and that food insecurity is related to future incidence of diabetes, even after taking into account differences in health behaviours, diet quality, and body mass index between respondents from food insecure versus food secure households.

The current study features many strengths. Much of the current literature on food security and type 2 diabetes has focused on differences in treatment and management characteristics (e.g. glycemic control, healthcare utilization) between food secure and food insecure individuals already diagnosed with diabetes. In contrast, our study addresses an important gap in identifying the long-term risk of type 2 diabetes associated with food security status. We use a validated measure for type 2 diabetes as an outcome, whereas much of the current literature has relied on self-reported diabetes status that is prone to underestimation. In a previous study comparing self-reported data from the Canadian Community Health Survey to the Ontario Diabetes Database, it was found that roughly one in four people with physician-diagnosed diabetes do not self-report having the disease [40], meaning that studies using self-reported diabetes as an outcome may be missing up to 25% of cases. Additionally, our sample was drawn to be representative of the adult population of Ontario, within which 40% of Canadians live allowing for inferences to be made at a provincial level. Whereas much of the current literature comes from cross-sectional studies which are limited in their ability to assess the direction of the association between food insecurity and type 2 diabetes, the prospective nature of our study over 12 years of follow-up in a population-based sample is an important strength. In particular, this limits the potential of the reverse causality hypothesis that suggests that diabetes may increase one’s awareness about inadequate healthy foods resulting in more conscious efforts to maintain food adequacy at the expense of other expenditures.

Despite these strengths, the results of our study should be interpreted with the consideration of a few important limitations. First, our outcome was limited to physician diagnosed diabetes. While the Ontario Diabetes Database features a highly sensitive (86%) and specific (97%) diagnostic algorithm, there is still a potential that some cases may not be captured, such as those with undiagnosed diabetes and those who do not have regular encounters with the healthcare system. However the prevalence of undiagnosed diabetes in Canada has been estimated at 1.13%, suggesting that failing to identify these individuals would not significantly impact the results [4]. Additionally, the ODD does not distinguish between type 1 and type 2 diabetes, however > 95% of diabetes cases are type 2 [41], and the present analyses were restricted to the adult population, reducing the likelihood of misclassification of diabetes type. Relatedly, our data did not allow for the ascertainment of whether individuals may have had a pre-diabetic condition at the outset of the study. Though individuals with pre-diabetes may go on to develop type 2 diabetes rendering this latent condition a potential source of bias, this suggests that our results may in fact be conservative as food insecure individuals would likely have a higher propensity for pre-diabetes. Further, there are no population-based cohorts in Canada that have the required blood measurement data necessary to assess pre-diabetes at baseline that could potentially mitigate this limitation. Second, household food insecurity may be underestimated because marginalized groups such as those living on First Nations reserves and homeless people are not included in the survey design, and these populations have an increased risk for developing diabetes [42]. However, given the data collection constraints pertaining to First Nations populations living on reserve in Canada, the design of CCHS did not allow for inclusion of information on this important subgroup of the population, and our results should be interpreted considering this limitation. Furthermore, we were unable to control for race/ethnicity in more detail than the binary operationalization presented due to small sample sizes in certain categories. Third, given the single measurement of food insecurity status at study baseline, we were unable to assess if food insecurity changed for some participants during the observation period. Fourth, although detailed survey data allowed for control of many potential confounders, there may be residual confounding due to the imprecision associated with self-reported data. However, CCHS has high quality data on covariates allowing for the simultaneous adjustment for factors associated with food security that are also known to influence type 2 diabetes risk. Furthermore, using the *Healthy Eating* Index, we adjusted for a validated measure of diet quality which is an important potential confounder that was uniquely able to be measured in CCHS 2.2 due to the detailed assessment of nutritional intake in this survey cycle. Lastly, our sample size for the current study was small resulting in a low number of food insecure individuals who were diagnosed with type 2 diabetes over 12 years of follow-up. However, the confidence intervals around the multivariable adjusted estimates we present reflect the precision allowed by this small sample size, and remained significant though investigating this relationship in a smaller, but still representative sample of the Ontario population.

To date, interventions addressing food insecurity have been developed at the community level, while a clear governmental policy to address food insecurity does not exist in Canada nor in the majority of developed countries [43]. Food banks and other charitable food providers that rely on donations from the public represent the only public source of food assistance for people who are experiencing or at risk of experiencing household food shortages. Many have highlighted the importance of food banks, affirming their role in addressing hunger and related health issues, and advocating that the strategic position that community organizations play in changing food insecurity intervention strategies may be strengthened [44, 45].

However, others have posited that food banks may exacerbate food insecurity by masking it and relieving the government of their duties [28, 46]. In this way, there is a very real concern that food banks, as the sole public response to problems of food insecurity and hunger, may be enabling the retraction of social programs [47]. Moreover, evidence has indicated that historically there has been low utilization of food banks among food insecure populations in Canada, suggesting that food banks have only ever served a small proportion of the growing number of Canadians struggling to get the quality food they need [16, 48].

In the United States, SNAP (Supplemental Nutrition Assistance Program) provides an example of a program that supports food security, has shown improvements in dietary intake in certain subgroups, and has shown to contribute to a lower risk of obesity. Increasing access to food and the implementation of food and nutrition assistance programs may offer a solution to mitigate the growing burden of food insecurity in Canada [49]. Such programs should seek to reduce the financial burden of healthy food by subsidizing fresh fruits and vegetables, rendering them a cheaper option than high carbohydrate, energy-dense food options associated with increased chronic disease risk [50].

Collaborating with existing community organizations and food retailers should be a priority for improving the availability of healthy food. For example, the provision of financial incentives to stores in low-income neighbourhoods to stock healthy low-cost foods, or to grocery store chains to open stores in food deserts could make inroads on reducing the number of Canadians living in food insecure households and the associated adverse health consequences [28].

The inadequacies of current social programs in developed countries have contributed to an upsurge in food insecurity over the past decade resulting in direct health implications. To date, no prospective studies have been published examining the association between food insecurity and type 2 diabetes in a population-based sample. Our findings support the need for interventions that actively pursue the objective of impacting the economic factors at the root of food insecurity, and the broader systemic factors that shape food production and distribution.

In recent years, most provinces and territories in Canada have initiated poverty reduction strategies, but social assistance reforms have not featured prominently in these strategies, and none has targeted reductions in food insecurity as an important outcome of their efforts [28]. As such, current directions in social policy and food bank operations provide no indications that this problem will correct itself in the near future.

Future policy directions should consider innovative programs that promote or support economic self-sufficiency for individuals, families, and households as a viable means to reduce food insecurity [51]. In the context of chronic disease prevention, further research is needed to determine the most effective strategies for counseling patients with limited financial resources to make healthy dietary changes [14]. For example, where food substitutions may be difficult, reducing portion size may be a more practical dietary strategy.

To better understand the mechanisms underlying the relationship between food insecurity and type 2 diabetes, future work should aim to further assess the role that obesity and chronic stress have in potentially mediating this observed association. Future research investigating whether interventions that support food security further reduce the population-level incidence of type 2 diabetes is also warranted. There is also a need to investigate the risk between food insecurity and overall, cause-specific, and premature mortality, in addition to other chronic disease outcomes such as cardiovascular disease, to comprehensively characterize the impact of food insecurity on leading causes of morbidity and mortality in developed countries.

## Supporting information

S1 Fig Study flow diagram. (PDF)

S1 Table Food security status classification. (DOCX)
