# Supplementary figures and images for: The association between food insecurity and incident type 2 diabetes in Canada: A population-based cohort study
Source: PLoS One. 2018 May 23;13(5):e0195962. doi: 10.1371/journal.pone.0195962 (PMC5965821; doi:10.1371/journal.pone.0195962)

Supplementary Figure 1. Study Flow Diagram

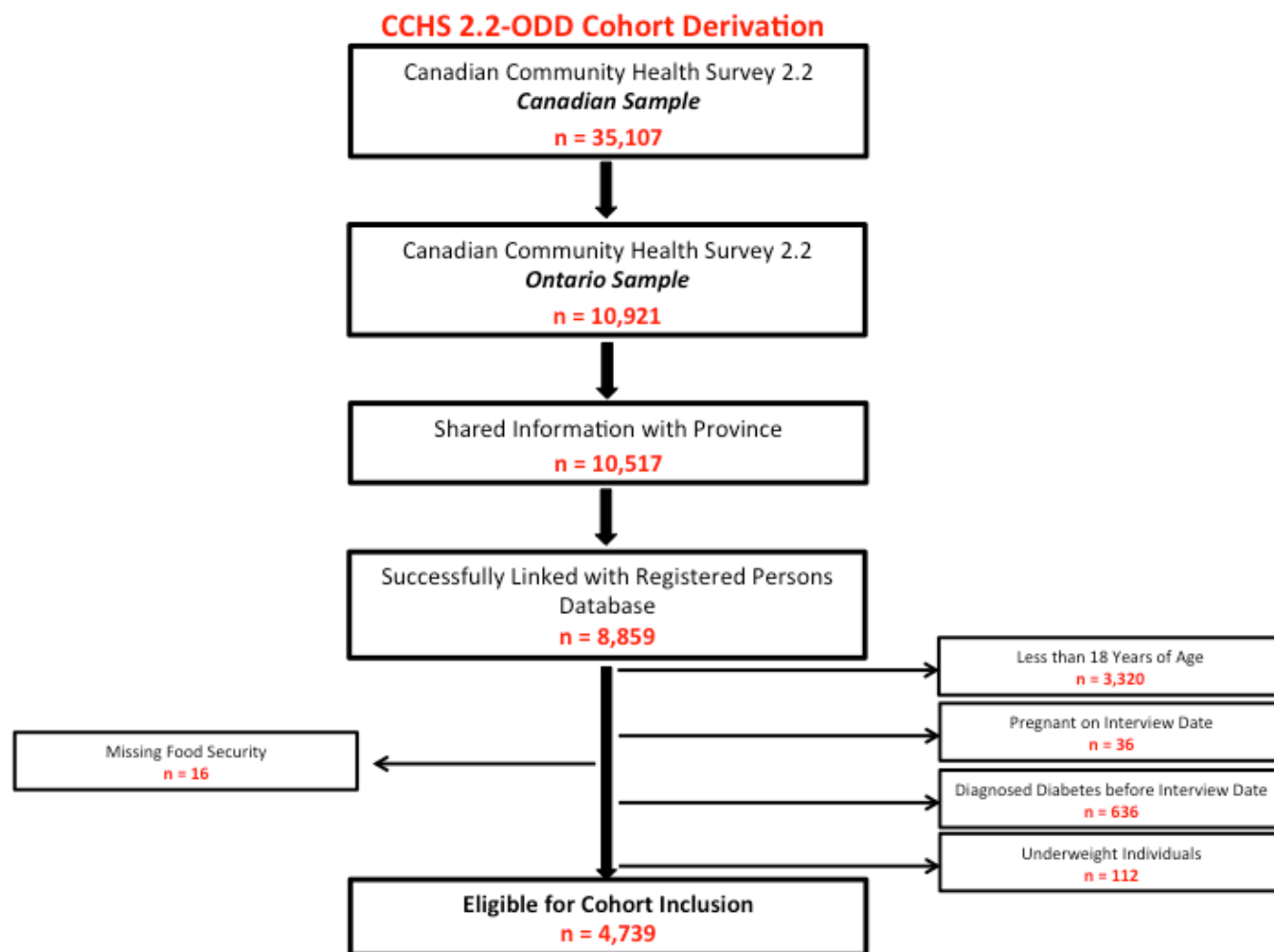

Supplement: S1 Fig — (PDF) [file pone.0195962.s001.pdf]
