# Supplementary material for: The association between food insecurity and incident type 2 diabetes in Canada: A population-based cohort study
Source: PLoS One. 2018 May 23;13(5):e0195962. doi: 10.1371/journal.pone.0195962 (PMC5965821; doi:10.1371/journal.pone.0195962)
Supplement: S1 Table — (DOCX) [file pone.0195962.s002.docx]

| **Food Security Status** | **Adult Status**  **(based on the Adult Scale)** | **Child Status**  **(based on the Child Scale)** | **Household Status**  **(derived from Adult and Child Status)** |
| --- | --- | --- | --- |
| Food Secure | No, or one indication of difficult with income-related food access  *0 or 1 affirmative responses* | No, or one indication of difficult with income-related food access  *0 or 1 affirmative responses* | Both adult status and child are food secure |
| Food Insecure | Indication of compromise in quality and/or quantity of food consumed or indication of reduced food intake and disrupted eating patterns  *≥ 2 affirmative responses* | Indication of compromise in quality and/or quantity of food consumed or indication of reduced food intake and disrupted eating patterns  *≥ 2 affirmative responses* | Either adults or children, or both adults and children in the household are food insecure |
